# Aging impairs double‐strand break repair by homologous recombination in *Drosophila* germ cells

**DOI:** 10.1111/acel.12556

**Published:** 2016-12-21

**Authors:** Laetitia Delabaere, Henry A. Ertl, Dashiell J. Massey, Carolyn M. Hofley, Faraz Sohail, Elisa J. Bienenstock, Hans Sebastian, Irene Chiolo, Jeannine R. LaRocque

**Affiliations:** ^1^ Molecular and Computational Biology DepartmentUniversity of Southern CaliforniaLos AngelesCA90089USA; ^2^ Department of Human ScienceGeorgetown University Medical CenterWashingtonDC20057USA; ^3^ College of Public Service & Community SolutionsArizona State UniversityPhoenixAZ85004USA

**Keywords:** aging, double‐strand break repair, *Drosophila melanogaster*, homologous recombination, Rad51

## Abstract

Aging is characterized by genome instability, which contributes to cancer formation and cell lethality leading to organismal decline. The high levels of DNA double‐strand breaks (DSBs) observed in old cells and premature aging syndromes are likely a primary source of genome instability, but the underlying cause of their formation is still unclear. DSBs might result from higher levels of damage or repair defects emerging with advancing age, but repair pathways in old organisms are still poorly understood. Here, we show that premeiotic germline cells of young and old flies have distinct differences in their ability to repair DSBs by the error‐free pathway homologous recombination (HR). Repair of DSBs induced by either ionizing radiation (IR) or the endonuclease I‐SceI is markedly defective in older flies. This correlates with a remarkable reduction in HR repair measured with the DR *‐white* DSB repair reporter assay. Strikingly, most of this repair defect is already present at 8 days of age. Finally, HR defects correlate with increased expression of early HR components and increased recruitment of Rad51 to damage in older organisms. Thus, we propose that the defect in the HR pathway for germ cells in older flies occurs following Rad51 recruitment. These data reveal that DSB repair defects arise early in the aging process and suggest that HR deficiencies are a leading cause of genome instability in germ cells of older animals.

## Introduction

Genome instability and DNA damage are hallmarks of old cells (reviewed in Gorbunova & Seluanov, 2016). More specifically, aging is accompanied by increase in chromosome rearrangements (Ramsey *et al*., 1995; Dolle *et al*., 1997; Tucker *et al*., 1999), oxidative DNA damage (Hamilton *et al*., 2001), and DNA double‐strand breaks (DSBs) (Sedelnikova *et al*., 2004). Similarly, cells from patients affected by premature aging syndromes, such as Hutchinson–Gilford progeria syndrome and restrictive dermopathy, are characterized by high level of DSBs (Liu *et al*., 2005). At the same time, mutations in several DSB repair components lead to premature aging syndromes, such as ataxia telangiectasia and Werner syndrome (Savitsky *et al*., 1995; Yu *et al*., 1996; Gorbunova & Seluanov, 2016), and DSB induction in mouse tissues leads to aging phenotypes (White *et al*., 2015). These studies suggest that DSB repair defects might be a driving force for aging. Whether the high level of DSBs detected in old cells represents higher incidence of damage or diminished capacity for repair as the organism ages is still unclear.

Most DSBs result from endogenous cellular by‐products, such as free radicals and single‐strand breaks, the latter of which are converted to DSBs during replication. Accurate repair of DSBs is accomplished by homologous recombination (HR), in which a homologous DNA sequence is used as a template to restore the information lost at the break. HR repair is initiated by 5′ to 3′ resection of the broken double‐strand, which is facilitated by the Mre11‐Rad50‐Nbs1 complex and CtIP, resulting in 3′ protruding single‐stranded DNA (ssDNA) (Chiolo *et al*., 2011; Nimonkar *et al*., 2011). Rad51 is recruited to this ssDNA substrate, and the resulting nucleoprotein filament guides homology search and strand invasion (Sugawara *et al*., 1995; McIlwraith *et al*., 2000). Invasion into the donor sequence on the sister chromatid or the homologous chromosome results in the formation of a D‐loop, after which Rad51 is removed to ensure DNA synthesis and HR progression (Williams & Michael, 2010). Components that facilitate Rad51 disassembly include yeast and human Rad54 (Li & Heyer, 2009; Wright & Heyer, 2014), and the *C. elegans* Rad51 paralog RFS‐1 and DNA helicase HELQ‐1 (Ward *et al*., 2010). After repair synthesis, the double‐Holliday junction (dHJ) and synthesis‐dependent strand annealing (SDSA) pathways of HR diverge. During dHJ, the D‐loop is extended to form two Holliday junctions that can be resolved into either a crossover or a non‐crossover product (Szostak *et al*., 1983). In contrast, during SDSA the newly synthesized strand of the D‐loop dissociates from the donor sequence and re‐ligates to the second end of the original DSB, resulting exclusively in a non‐crossover product. Meiosis relies primarily on dHJ repair, while SDSA is the preferred pathway for mitotically dividing cells, and both pathways are largely error‐free. Furthermore, both homologous chromosomes and sister chromatids can be used as templates for repair, but the sister chromatid is the preferred template in S/G2 cell cycle phases of mitotically dividing cells (Rothkamm *et al*., 2003; Janssen *et al*., 2016). While several studies revealed that error‐prone DSB repair pathways become defective with age (Ren & Pena de Ortiz, 2002; Seluanov *et al*., 2004; Preston *et al*., 2006a; Vaidya *et al*., 2014), the impact of aging on HR in cultured cells and *in vivo* is still controversial (Hendricks *et al*., 2003; Preston *et al*., 2006b; Mao *et al*., 2012; White *et al*., 2013; Sukup‐Jackson *et al*., 2014).

Premeiotic cells of *Drosophila melanogaster* are an excellent system to study the effects of aging on HR. These dividing cells of adult flies are subjected to age‐related changes and mortality (Wallenfang *et al*., 2006), they largely rely on HR for DSB repair (Rong & Golic, 2003; Chan *et al*., 2011), and repair outcomes are easily detectable in the progeny (Rong & Golic, 2003; Preston *et al*., 2006b; Johnson‐Schlitz *et al*., 2007; Chan *et al*., 2011; Do *et al*., 2014). Further, those are arguably among the most important cells of adult organisms as their genome integrity is necessary for producing viable and healthy progeny. Lastly, the male germline develops early during embryogenesis and begins meiosis at pupariation (Lindsley, 1976). Thus, spermatogonia are fully developed by eclosure of adults, providing an ideal cell population to study the effects of aging on DSB repair starting from very ‘young’ adults – as early as 1 day old (d.o.) (Boyle *et al*., 2007; Toledano *et al*., 2012).

Previous studies in Drosophila premeiotic germ cells suggested that interhomolog HR repair is more efficient in older flies, potentially ruling out defective HR as a contributor to genome instability and repair defects in aging (Preston *et al*., 2006b). This study also demonstrated that ‘error‐prone’ repair pathways (*i.e.,* nonhomologous end joining (NHEJ) and single‐strand annealing (SSA)) prevail in young flies, suggesting that, unexpectedly, DNA repair becomes more accurate as the organism ages (Preston *et al*., 2006b).

While we were able to recapitulate these results in a model of constitutive DSB induction, here we challenge this larger conclusion, showing that aging results in a remarkable defect in repairing ionizing radiation (IR)‐ or endonuclease‐induced DSBs in spermatogonia. Strikingly, induction of DSBs in the DR‐*white* reporter (Do *et al*., 2014) at various ages reveals a significant decrease in intrachromosomal or intersister chromatid HR in the spermatogonia of older organisms (8 d.o. and older), revealing a very early effect on the proficiency of HR repair. Interestingly, this defect correlates with higher expression of early HR components, and increased Rad51 recruitment to repair sites. This not only suggests that HR defects may be caused by deregulation of repair steps following Rad51 recruitment, but also that excessive Rad51 recruitment might contribute to this phenotype. Contrary to previous conclusions, this study uncovers a dramatic effect of aging in the ability of premeiotic cells to complete HR repair, suggesting HR deregulation as a major source of age‐dependent genomic instability and cell lethality in the male germline.

## Results

### Aging results in defective repair of IR‐induced DSBs in premeiotic germ cells

**Figure 1:**
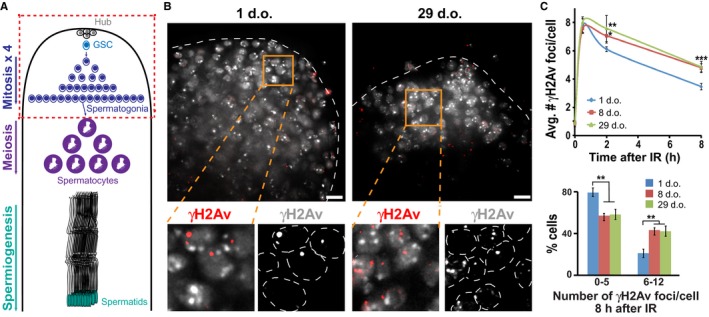
DSB repair is delayed in older animals. (A) Schematic view of Drosophila spermatogenesis, indicating the position of premeiotic cells in the testis (red rectangle) corresponding to the top images in B. GSC = germline stem cell. (B) Immunofluorescence (IF) analysis of premeiotic cells in testes dissected and fixed 8 h after 5 Gy IR shows more γH2Av foci in mitotically dividing spermatogonia of 29‐day old (d.o.) flies compared to those of 1‐d.o. flies. Scale bars = 5 μm. (C) Quantification of γH2Av foci in spermatogonia shows higher average number of γH2Av foci at 2 and 8 h after IR (top) and higher frequency of cells with more foci at 8 h after IR (bottom) in older flies relative to young flies. Error bars: SD. Top: *P = 0.04 (one‐tailed Mann–Whitney test); **P = 0.0056 and ***P < 0.001 (two‐tailed Mann–Whitney test). Bottom: **P < 0.005 (Welsh's test). All p values refer to comparisons with 1‐d.o. flies. Differences between 8‐ and 29‐d.o. flies were not significant. *n* = 85–168 nuclei from at least three independent testes/age/time point.

We investigated the efficiency of DSB repair in premeiotic germ cells (Fig. 1A) of male flies during aging, by determining the kinetics of DSB formation and resolution following IR (Chiolo *et al*., 2011). Testes were dissected and fixed at different time points after IR, and stained for γH2Av foci, a marker for DSBs (Fig. 1B; Chiolo *et al*., 2011). Quantification of γH2Av focus number indicates that DSBs form with similar kinetics in newly emerged (1 d.o) and older (8 d.o. and 29 d.o.) flies (Fig. 1C, time point 30 min). However, older flies display a higher number of γH2Av foci at both 2 and 8 h after IR, relative to 1‐d.o. flies, revealing defects in DSB resolution (Fig. 1B,C). Notably, there is no difference between young and old flies in the number of repair foci before IR (Fig. 1C, time point 0 h). This suggests that ‘spontaneous’ DSBs do not accumulate with age in these cells and do not significantly contribute to the higher number of ‘persistent’ DSBs observed in older flies. Similar repair defects were observed when Mu2/Mdc1 foci were used as a marker for DSBs (Dronamraju & Mason, 2009; Chiolo *et al*., 2011; Fig. S1, Supporting information). Further, repair defects are already present in 8‐d.o. flies (with intermediated levels detected in 5‐d.o. flies; Fig. S1, Supporting information), but no significant differences are observed between 8‐ and 29‐d.o. flies (Fig. 1C). We conclude that aging compromises DSB repair efficiency in premeiotic cells of adult flies and that this effect arises early on during the aging process.

### HR repair of premeiotic germ cells becomes defective as the organism ages

**Figure 2:**
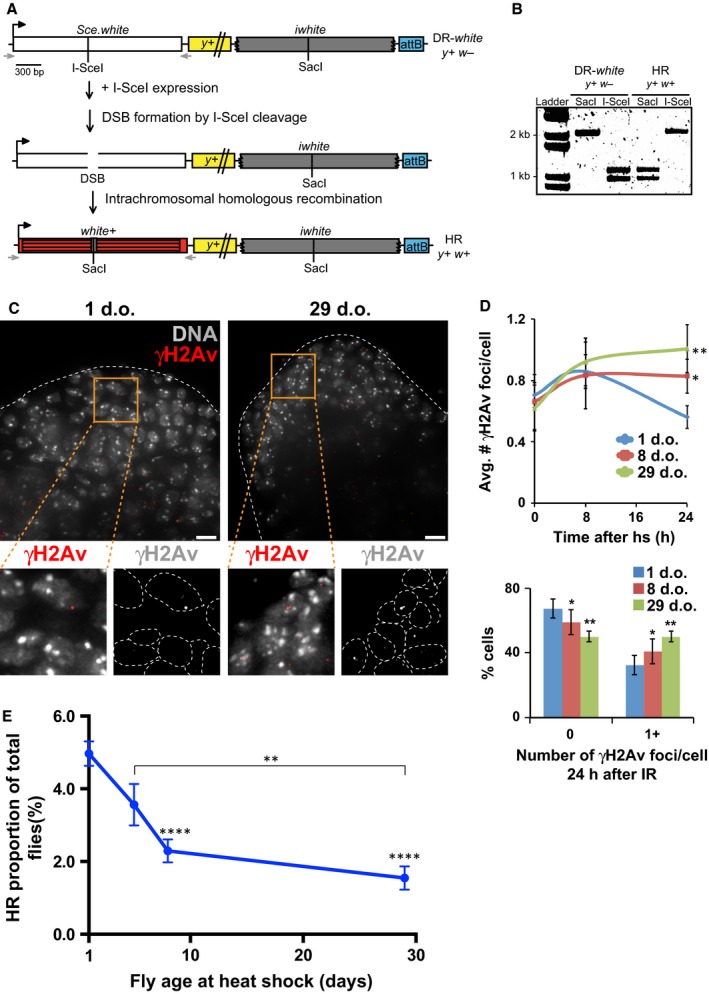
HR repair of I‐SceI induced DSBs decreases with age. DSB repair is measured by I‐SceI induced DSBs using the DR‐*white* reporter. (A) The DR‐*white* assay contains two nonfunctional direct repeats of the *white* gene. The first repeat, *Sce.white*, is nonfunctional due to the insertion of an I‐SceI recognition sequence into the wild‐type *white* cDNA. This results in a premature STOP codon. The second repeat, *iwhite*, is nonfunctional due to 5′ and 3′ truncations, but contains wild‐type *white* sequence, including a SacI cut site, at the location correspondent to the I‐SceI site in *Sce.white*. DR‐*white* flies are crossed with flies containing the I*‐*SceI transgene, which results in DSB formation at the I‐SceI recognition sequence. Repair by HR results in restoration of the wild‐type sequence and a red‐eyed fly (*y* ^*+*^ *w* ^*+*^) in the progeny. (B) HR repair resulting in gene conversion of the wild‐type SacI sequence can be confirmed molecularly. *Sce.white* gene is amplified using primers indicated in (A) (gray arrows), followed by digestion of PCR product with SacI or I‐SceI. I‐SceI cleaves only intact *Sce.white* sequence. SacI cleaves only HR products. (C‐D) Flies containing the DR‐*white* chromosome and the hs‐I‐SceI transgene were heat‐shocked at the indicated ages. Testes were dissected at given time point and stained for γH2Av foci. (C) IF analysis shows more γH2Av foci in spermatogonia of 29‐d.o. flies compared to 1‐d.o. flies, at 24 h after heat shock. Scale bars = 5 μm. (D) Quantification of γH2Av focus number in spermatogonia fixed prior to and at different time points after heat shock shows higher number of γH2Av foci (top) and higher frequency of cells with one or more foci (bottom) in 8‐ and 29‐d.o. flies relative to 1‐d.o. flies, at 24 h after heat shock. Error bars: SEM; *P < 0.05 and **P = 0.005 by two‐tailed Mann–Whitney test. p values refer to comparisons with 1‐d.o. flies. Differences between 8‐ and 29‐d.o. flies were not significant. *n* = 38–167 nuclei from at least two independent testes/age/time point. (E) Flies containing the DR‐*white* reporter and hs‐I‐SceI transgene were aged to the given times and exposed to heat shock. After 11 days, flies were mated to females and F1 progeny scored for HR products. Data given are mean ± SEM of 73–123 germlines. **P < 0.01; ****P < 0.0001 for comparisons to 1 d.o., by one‐way ANOVA with multiple comparisons followed by Tukey–Kramer post hoc test. Differences between all other ages were not significant.

To investigate whether the repair defects observed in older flies are a consequence of defective HR, we analyzed repair outcomes with the DR‐*white* repair reporter assay (Do *et al*., 2014). In this system, two nonfunctional repeats of the *white* gene (*Sce.white* and *iwhite*) are inserted in a euchromatic locus on Chromosome 2 (Fig. 2A). Exposure to the I‐SceI meganuclease results in specific cleavage of both strands of the DNA in *Sce.white,* causing a DSB. Intrachromosomal or intersister chromatid HR repair using the *iwhite* sequence as a template restores the wild‐type SacI sequence of the *white* gene, resulting in red eyes in the progeny (Fig. 2A,B).

We first confirmed that within the DR‐*white* system, I‐SceI induces DSBs with similar efficiency in young and older flies by investigating repair focus kinetics. To induce DSBs at specific ages, adults that contain both the DR‐*white* reporter and a heat‐shock‐inducible I‐SceI transgene (hs‐I‐SceI) were aged and then heat‐shocked. Quantification of γH2Av foci in the premeiotic male germ cells revealed that after heat shock, focus numbers are initially similar in young and older flies (Fig. 2C,D, time point 8 h). This suggests that I‐SceI induction and DSB formation are equally efficient at different ages of the fly, validating the use of the DR‐*white* system for aging studies. We further observed that the γH2Av focus number decreases over time in young flies (1 d.o.), reflecting repair progression (Fig. 2D, time point 24 h). Conversely, γH2Av focus number remains high in older flies (Fig. 2C,D). We conclude that older animals (≥ 8 d.o.) have a reduced ability to repair I‐SceI‐induced DSBs in their germline, similar to their defect in repairing IR‐induced DSBs.

Next, we investigated whether repair defects in germ cells of older animals are a consequence of defective HR, by measuring repair outcomes in the progeny. Adult flies containing both the DR‐*white* chromosome and hs‐I‐SceI were aged to 1, 5, 8, and 29 days and then heat‐shocked. These males were crossed with control females 11 days later, allowing time for the spermatogonia affected by DSB induction and repair to develop into mature sperm (Lindsley, 1976). Notably, this timing corresponds to the peak of HR events detectable in the progeny (data not shown), consistent with the fact that HR is the main DSB repair pathway used in premeiotic cells but not in later differentiation stages (Rong & Golic, 2003; Preston *et al*., 2006a; Chan *et al*., 2011). A progressive decrease in the proportion of red‐eyed progeny was observed between 1 and 8 days, revealing that the frequency of repair by HR quickly declines with age (Fig. 2E). Similarly to γH2Av focus kinetics, there is not a significant difference between 8‐ and 29‐day‐old flies (Fig. 2E). Notably, spontaneous HR repair events do not significantly change across different ages (Table S1, Supporting information), suggesting that the decrease in proportion of red‐eyed progeny in older flies is due specifically to defects in HR repair of I‐SceI‐induced DSBs. In contrast to HR defects, we did not detect SSA defects in 8‐ to 29‐d.o. animals relative to 1‐d.o. animals (Fig. S2, Supporting information). We note, however, that SSA events detected with the DR‐*white* system are rare (about 10‐fold less relative to HR (Do *et al*., 2014)); thus, small changes in this repair pathway may be below detection level.

Importantly, these results are in striking contradiction with the previously reported increase in HR repair in old flies (Preston *et al*., 2006b). This discrepancy might depend on the different reporter assay (interhomolog HR vs. intrachromosomal or intersister‐chromatid HR) or the constitutive expression of I‐SceI used in previous experiments. We directly tested the second possibility by repeating the DR*‐white* repair assay in flies of different ages with constitutive expression of I‐SceI. In agreement with previous studies, constitutive I‐SceI expression leads to higher levels of HR products detected in the progeny as the organism ages (Fig. S3, Supporting information). This points to the constitutive vs timed activation of I‐SceI as the most likely explanation for the differences observed in the two studies. We suggest that constitutive expression of I‐SceI across development and differentiation favors the detection of HR products in the progeny, potentially skewing the data toward HR outcomes (see Discussion). Together, our data suggest that, contrary to previous conclusions, aging leads to a marked defect in HR repair of DSBs in premeiotic male germ cells.

### HR repair defects in older animals correlate with increased expression of early HR components and Rad51 localization to repair foci

To gain insight on the mechanism causing HR defects in older animals, we investigated how aging affects the expression of HR genes, particularly at the ages where the proportion of HR progeny rapidly declines (1, 5, and 8 d.o). Flies containing both DR‐*white* and hs‐I‐SceI transgene were aged for 1, 5, and 8 days and then harvested. We analyzed the mRNA expression of genes required for different HR steps, particularly DSB detection and resection (*rad50*,* ctip/CG5872,* and *blm*), strand invasion (*rad51/spnA*), and D‐loop processing (*rad54*/*okr*).

**Figure 3:**
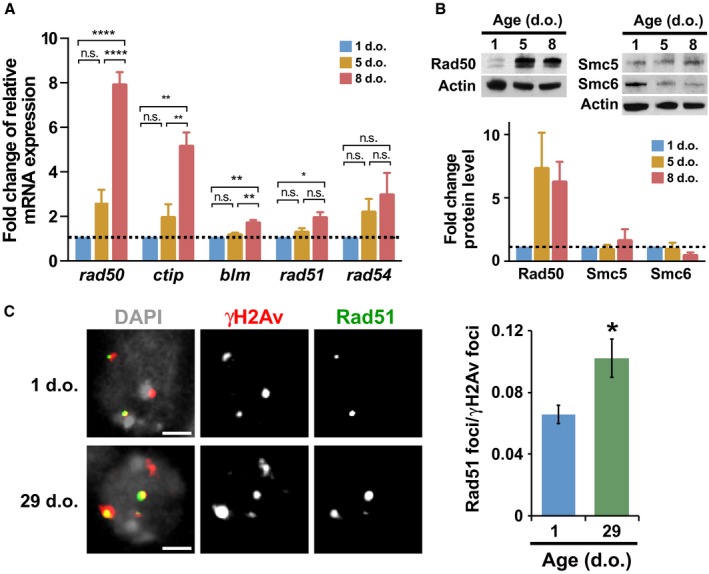
Expression of early HR components, and Rad51 localization to DSBs, increase in older animals. Flies containing the DR‐*white* reporter and the hs‐I‐SceI transgene were aged to the given times. (A) C_T_ values for *rad50, ctip, rad51,* and *rad54* were normalized to that of *gapdh2* (C_T_) to determine relative expression. ΔΔC_T_ values were calculated relative to 1‐d.o. flies for each gene to determine expression fold change (2^‐ΔΔCT^). Averages of the fold change are given; error bars are SEM of 3–10 biological replicates. *P < 0.05; ** *P* < 0.01; *** *P* < 0.001; **** *P* < 0.0001, by one‐way ANOVA with multiple comparisons, followed by Tukey‐Kramer post hoc test. (B) Western blot analysis of protein extracts from male flies of different ages shows Rad50, Smc5, and Smc6 protein levels relative to 1‐d.o. flies. Actin is used as loading control. Error bars: SD; *n* = 3 independent experiments; representative blots are shown. (C) Left: IF analysis of Drosophila testes dissected and fixed 4 h after 5 Gy IR shows examples of Rad51 foci colocalizing with γH2Av foci in mitotically dividing spermatogonia of 1‐ and 29‐day‐old (d.o.) flies. Scale bars = 1 μm. Right: Quantification of the ratio of Rad51 foci colocalizing with γH2Av foci over total γH2Av foci in spermatogonia shows an increase in older flies (29 d.o.) compared with young flies (1 d.o.). Error bars: SD; *P = 0.0166, Mann–Whitney test. *n* = 125 nuclei for 1‐d.o. flies and *n* = 116 nuclei for 29‐d.o. flies from at least three independent testes/age.

Interestingly*,* we found statistically significant increases in relative mRNA expression levels of *rad50, ctip, blm,* and *rad51,* in 8‐d.o. flies relative to both 1‐d.o. and 5‐d.o. flies (Figs 3A and S4A, Supporting information). mRNA expression levels of *rad51* remain elevated in 15‐ and 29‐d.o. flies (Fig. S4B, Supporting information). Accordingly, Rad50 protein levels dramatically increase in older flies (Fig. 3B). These responses were not observed for all HR genes, as Smc5 or Smc6 protein levels did not display major changes between 1‐ and 8‐d.o. flies (Fig. 3B). Changes in rad54 mRNA levels were also not significant (Fig. 3A), possibly reflecting high variability in rad54 expression in older flies.

Notably, Rad51 overexpression has been previously linked to HR defects, aberrant recombination, and genome instability (Richardson *et al*., 2004; Paffett *et al*., 2005; Klein, 2008), as high levels of this protein might impede HR progression by interfering with Rad51 displacement after strand invasion. In agreement with this prediction, older flies display a higher number of Rad51 foci colocalizing with γH2Av foci at 4 h after IR (Fig. 3C). Given that Rad51 focus formation is not reduced in older flies, we conclude that early HR steps (resection and Rad51 recruitment) are not negatively affected by aging. Rather, we propose that the observed HR defects are caused by malfunctions of later steps. Specifically, overexpression of resection components and Rad51 might contribute to HR defects by overloading Rad51 and hindering the disassembly of the nucleoprotein filament after strand invasion.

## Discussion

Many factors contribute to aging and this biological process encompasses all systems, ranging from the cellular level to the organismal level. One hallmark of aging is an increase in genome instability, as evidenced by higher levels of DNA damage and tumorigenesis in natural aging, and by the high level of genome instability characterizing premature aging syndromes. However, the mechanisms leading to genome instability in aging organisms remain unclear. Here, we have utilized Drosophila to measure the effects of physiological aging on HR repair at the cellular level within the context of aging organisms. We found that IR‐ and endonuclease‐induced DSBs persist in premeiotic germline cells of older animals and that repair by intrachromosomal or intersister HR becomes significantly defective in the mitotically dividing spermatogonia as the organism ages. Pronounced HR defects appear early in aging organisms, and excessive resection and/or the accumulation of Rad51 protein onto resected DSBs might contribute to these defects. We propose that high levels of genome instability that characterize older animals are, at least in part, a consequence of a decreased ability to repair DSBs through error‐free HR repair.

Data demonstrating increased γH2AX staining in older mammalian cells (Sedelnikova *et al*., 2004) concluded that DNA damage accumulates over time, which might reflect a combination of higher levels of spontaneous damage from oxidative reactions (Hamilton *et al*., 2001) and/or defective repair (Gorbunova *et al*., 2007; Gorbunova & Seluanov, 2016). However, in our experiments the baseline level of spontaneous DSBs in premeiotic cells is not significantly different between young and old flies, enabling a more direct evaluation of changes in repair efficiency. Further, shortly after DSB induction with IR or I‐SceI, the frequency of γH2Av foci was similar regardless of age, suggesting that early repair steps are not compromised in older flies. This also argues against major defects in DSB formation, or in DSB repair by NHEJ which typically occurs within ~30 min after IR (Mao *et al*., 2008). Rather, the persistence of γH2Av foci in older animals suggests a specific defect in DSB repair by slower pathways, such as HR.

The DR‐*white* system allows us to directly test if DSB repair by intrachromosomal or intersister chromatid recombination is affected with age. Using a constitutively active I‐SceI enzyme, the frequency of HR repair increases over time. In agreement, Preston, *et al*. showed that constitutive induction of I‐SceI in a Rr3 reporter leads to more interhomolog HR with age (Preston *et al*., 2006b). However, several caveats to these experiments warrant a guarded approach in interpreting these results. First, in both the Rr3 and the DR‐*white* systems, HR repair of DSBs results in the loss of the I‐SceI recognition sequence, while simple NHEJ without processing will restore the cleavable sequence. This intrinsically biases the observed output toward the terminal repair event that cannot be further cleaved by I‐SceI (such as HR). Thus, HR outcomes may be more frequently detected when I‐SceI is constitutively expressed, and the more prolonged expression of I‐SceI in older flies might explain the observed increase in HR products in the progeny. Second, constitutive DSB formation may result in a DNA damage response unique to this system where repeated break events occur at the same site. Last, constitutive DSB formation in the germline may eventually impact the pool of germline stem cells. Repair of a stem cell by HR would result in 100% of the progeny arising from that stem cell displaying the HR product phenotype for the remainder of the life of the male, resulting in an overall increase in observed HR frequency in the progeny population. In fact, this was observed on several occasions, where by 29 days of age, all of the progeny from a single germline were HR events (data not shown).

**Figure 4:**
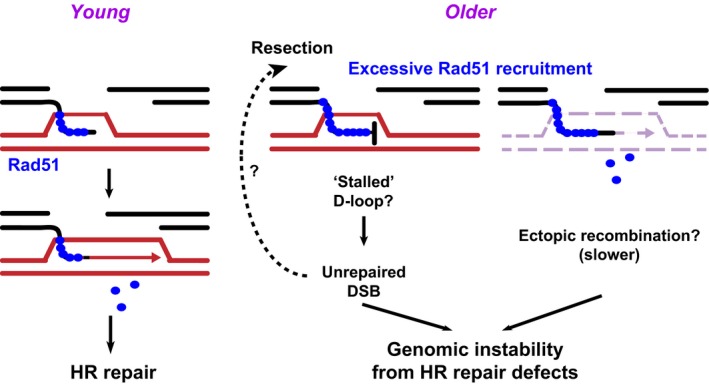
Model for HR defects in aging. In older flies, Rad51 overexpression and persistence at DSBs correlates with defective HR progression. The increased expression of resection components might aggravate this response by enhancing Rad51 nucleofilament formation. We propose that excessive Rad51 recruitment may counteract nucleofilament disassembly, resulting in persistent unrepaired DSBs and chromosome rearrangements. Excessive Rad51 may also trigger ectopic recombination (chromosome exchanges with nonhomologous chromosomes), which also typically occurs with delayed kinetics (Chung *et al*., 2010), and contributes to ‘persistent’ damage foci and genome instability.

Due to these concerns, we used an inducible system to generate DSBs within a narrow timeframe, and the reproduction was timed so that observed progeny reflected the outcomes of repair within the premeiotic cell population. In contrast to what is observed in the constitutively active system, the heat‐shock‐induced DSBs were repaired by HR at decreased frequencies in older animals. Because of the ability to induce damage at a specific time point in the organism's lifespan, and because we observed similarly low baseline levels of damage across age groups, we suggest that this assay more accurately represents how DSBs are repaired in the spermatogonia of older animals. In agreement with our analysis of repair focus kinetics, these results suggest that defective HR repair significantly contributes to repair defects in older germ cells. Interestingly, HR defects have also been detected in presenescent human fibroblasts, an *in vitro* model of aging (Mao *et al*., 2012). Similarly, interchromosomal HR defects occur in somatic cells of older mice (White *et al*., 2013), suggesting that recombination defects might be a common problem in older cells. While more studies are required to directly address the effects of aging on interhomolog HR repair, our study is the first direct demonstration that intrachromosomal or intersister chromatid HR repair of DSBs induced in the germline of older animals declines, supporting the conclusion that HR defects may promote genome instability in older organisms (Fig. 4).

What can be the cause of HR defects in older flies? Previous studies in presenescent human cells identified a reduction of Rad51 levels and focus formation, suggesting Rad51 recruitment as a limiting step for HR in old cells (Mao *et al*., 2012). However, neither Rad51 expression nor recruitment to repair foci is defective in the spermatogonia of older flies, suggesting that early HR steps are proficient in this context. This difference between presenescent human cells and aging flies suggests the interesting possibility that deregulation of more than one recombination step might contribute to HR defects during aging. One possibility is that the abnormally high levels of Rad51 in older flies interfere with Rad51 disassembly postsynapsis and repair completion (Fig. 4). Accordingly, we observed a greater frequency of Rad51 foci associated with γH2Av foci in older flies, possibly reflecting a defect in disassembling the nucleoprotein filament after strand invasion. Additionally, the increase in interhomolog HR observed by Preston *et al*. was associated with an increase in longer gene conversion tracts (Preston *et al*., 2006b), and defective Rad51 disassembly may contribute to this phenotype. What participates in the nucleofilament disassembly in Drosophila is still unknown, but homologues of Rad54 (*okra*) (Kooistra *et al*., 1999), HELQ‐1 (*mus301*) (McCaffrey *et al*., 2006), and several Rad51 paralogs exist in flies (Morris & Lehmann, 1999) and might play conserved roles in Rad51 displacement from the postsynaptic filament. Rad51 overexpression and persistent nucleofilaments could also induce ectopic recombination, further promoting genome rearrangements (Richardson *et al*., 2004; Klein, 2008), particularly if suppressors of Rad51‐mediated aberrant recombination (*e.g.,* Smc5/6, Chiolo *et al*., 2011; Li *et al*., 2013) are not equally induced. Notably, the higher expression of genes required for early repair steps might aggravate these effects by channeling DSBs toward the HR pathway and/or by over‐resecting the DSBs.

While the cause of Rad51 overexpression is still unclear, high Rad51 levels have been proposed as a leading cause of chromosome aberrations in cancer cells (Maacke *et al*., 2000; Raderschall *et al*., 2002; Richardson *et al*., 2004). Our current data do not directly determine whether increased recruitment of Rad51 is a cause or consequence of defective HR. However, we propose that abnormally high expression of resection components and Rad51 might be a driving force for genome instability and cancer formation in old organisms and future studies will directly test this possibility.

In all our experiments, the most significant differences in HR repair were observed when comparing 1‐ and 8‐day‐old animals. These data suggest that the effects of aging on DSB repair are not linear with age. Rather, there may be a threshold that regulates HR repair efficiency in young animals, and once this is reached (for example, between 5 and 8 days), this efficiency is lost. Considering the median and mean lifespan of male Drosophila in standard culturing conditions in the laboratory is ~50 days (Linford *et al*., 2013), the age at which HR repair capacity decreases is relatively young. This may have implications for other organisms as well, suggesting that HR repair is defective even before ‘old age’. Together, our data revealed a striking defect in error‐free HR repair in premeiotic germ cells of older animals, suggesting HR defects as a cause of the characteristic cancer predisposition, infertility, and developmental defects in the progeny, observed in older organisms.

## Experimental procedures

### Drosophila stocks and maintenance

Drosophila were maintained on standard media at 25 °C, which was either Nutri‐fly Bloomington Formulation medium (Genesee Scientific; San Diego, CA, USA) or prepared as in (Ren *et al*., 2009). I‐SceI transgenic stocks included either a ubiquitin promoter for constitutive active expression (Preston *et al*., 2006a) or hsp70 promoter for heat‐shock induction (Rong & Golic, 2003). Standard genetic crosses were used to create DR‐*white*/I‐SceI males in a *y w* background. *y ry* or mGFP‐Mu2‐expressing flies were used in ionizing radiation experiments. For aging time points, flies were collected for 24 h before treatment or aging; thus, 1 d.o. age corresponds to flies 0–1 day old, as in previous studies (Boyle *et al*., 2007; Toledano *et al*., 2012). Similarly, 5 d.o. refers to 4‐ to 5‐d.o. flies, and so on. mGFP‐Mu2‐expressing flies are a kind gift from J. Mason (Dronamraju & Mason, 2009).

### Immunofluorescence and imaging of male germline

For IR experiments, flies were exposed to 5 Gy using a 160‐kV X‐ray source (X‐RAD iR‐160, Precision X‐Ray) before testis dissection. For staining post‐I‐SceI induction, male testes were dissected and fixed at given time points after heat shock. Notably, I‐SceI induction requires more time to induce DSB formation relative to IR (compare Figs 2D to 1C). This is likely because of the time required to induce I‐SceI expression, which results in less synchronous DSB formation in the cell population (Janssen *et al*., 2016). Testes were dissected in PBS–Triton 0.1% (PBST) and fixed at room temperature in 4% paraformaldehyde in PBST for 8 min, rinsed three times in PBST, and blocked for 1 h at room temperature with milk 4% in PBST. Primary antibodies were incubated overnight at 4°C, and secondary antibodies were incubated at RT for 1 h, both in the blocking solution. Antibodies were as follows: anti‐γH2Av (Rockland, Cat. # 600‐401‐914, 1:1000), anti‐Rad51/SpnA (kind gift from J. Kadonaga, 1:1000), and anti‐GFP (Aves Labs, Cat #GFP‐1020, 1:500). For Rad51 and γH2Av colocalization experiments, primary antibodies were directly labeled with Alexa‐488 (Rad51) and Alexa‐555 (γH2Av) as described in Oegema *et al*. (2001). DNA staining, slide mounting, imaging with a DeltaVision microscope (Applied Precision/GE Healthcare), and image analysis with the softWoRx software were performed as previously described (Chiolo *et al*., 2011; Ryu *et al*., 2015).

### Measuring HR and SSA repair with DR‐white assay

DR‐*white* was targeted in the genome using the attB sequence, and integration was confirmed using *yellow* (*y+*) transgene expression (Do *et al*., 2014). HR and SSA repair of I‐SceI‐induced DSBs in DR‐*white* was measured as previously described (Do *et al*., 2014). Briefly, I‐SceI expression results in cleavage of the I‐SceI recognition sequence in *Sce.white*. DSBs occur in the somatic and germline cells. To isolate single repair events and determine HR and SSA repair frequency, males containing repair events in their germline are crossed to *y w* virgin females. Repair by HR includes utilizing the downstream *iwhite* sequence restoring the wild‐type *white* sequence, which results in wild‐type *white* expression and red‐eyed progeny. Gene conversion of the wild‐type *iwhite* sequence is confirmed molecularly by amplification of the repair events as described previously (Do *et al*., 2014). Briefly, *Sce.white* was PCR‐amplified using *Sce.white*‐specific primers (Fig. 2A) (forward, 5′ GTTTTGGGTGGGTAAGCAGG; reverse, 5′ AGACCCACGTAGTCCAGC) using SapphireAmp Fast PCR Master Mix (Clontech, Mountain View, CA, USA). For I‐SceI and SacI digests, PCR products were directly digested (New England Biolabs, Ipswich, MA, USA). Repair by SSA results in loss of the *yellow* (y+) transgene. NHEJ with processing (e.g., loss of I‐SceI recognition sequence) products detected with the DR‐*white* system was negligible (data not shown).

### *DR‐*white *aging experiments*

To obtain flies with inducible DSBs for aging, females containing the DR‐*white* reporter were crossed to males containing the heat‐inducible I‐SceI transgene (Wei & Rong, 2007) in large embryo collection cages in 24‐h increments to synchronize eclosion. Progeny of this cross, containing DR‐*white* and heat‐shock‐inducible I‐SceI transgene, were collected for 24‐h increments and then aged to given time points. To minimize losses while aging, 30 flies/vial were flipped onto fresh food twice a week.

For 5‐, 8‐ and 29‐d.o. experiments, male flies were outcrossed to *y w* virgins at a 1:1 ratio for 5 days (3 days for 5‐d.o. males) prior to heat shock to reset their germline. Females were removed 24 h before heat shock. Flies were then heat‐shocked at 37.5 °C for 36–38 min. To capture only repair events in the premeiotic germline, 1, 5, 8, and 29‐d.o. heat‐shocked males were aged for an additional 11 days at the conditions mentioned above and single males were crossed to 5–8 *y w* virgins. Progeny of this cross were scored. Only vials containing ≥ 20 progeny were included in analyses. Each vial represented one germline (*n*). Notably, flies scored for HR defects are expressed as a percentage of the total progeny; thus, potential fluctuations in the number of spermatogonia (and total progeny) with age are unlikely to contribute specifically to changes in HR frequencies.

To analyze accumulating repair events in aged animals with constitutively active I‐SceI transgene, aging experiments were performed similar to Preston, *et al*. (Preston *et al*., 2006b). Briefly, single male flies containing both DR‐*white* and the I‐SceI transgene constitutively activated by the ubiquitin promoter (Preston *et al*., 2006a) were crossed to new 4–5 *y w* virgins at 7‐day intervals. After 7 days, the parents were removed; females were discarded and males were crossed to 5 new *y w* females. Only males that survived and had productive progeny (≥ 20 progeny) at all four ages were included in analyses. Each vial represented one germline (*n*).

### qPCR analysis of genes in HR pathway

Male flies containing both DR‐*white* and the hs‐I‐SceI transgene were synchronized and aged as above to 1, 5, and 8 days. Two flies were combined to represent one biological replicate, and five biological replicates per age were harvested. mRNA was purified by acid guanidinium thiocyanate–phenol–chloroform extraction with TRIzol^®^ LS (Invitrogen; Carlsbad, CA, USA) and RNA Clean and Concentration‐5 (Zymo Research; Irvine, CA, USA). Contaminating DNA was removed by DNA‐*free*™ rDNase I treatment (Invitrogen). Reverse transcription was performed using SuperScript™ VILO Master Mix (Invitrogen), and quantitative real‐time polymerase chain reaction (qPCR) was primed with the RT^2^ SYBR Green Master Mix, using RT^2^ qPCR Primers for *Drosophila melanogaster rad51, rpl32, rad50, CG5872 (ctip), gapdh2, and rad54* (cat. n.:(PPD10573A, PPD10569B, PPD05479A, PPD07756A, PPD01317A, and PPD02120A, respectively, from Qiagen; Hilden, Germany). *gapdh2* and *rpl32* were used for normalization due to low expression variation with age (Ling & Salvaterra, 2011). All qPCR measurements were obtained using the 7900HT Real‐Time Thermal Cycler and software (Applied BioSystems; Carlsbad, CA). C_T_ values for each experimental gene were normalized to that of *gapdh2* or *rpl32* (C_T_) to determine relative expression. ΔΔC_T_ values were calculated relative to 1‐d.o. flies within each experiment for each gene to determine expression fold change ($2^{-\Delta \Delta C_{T}}$). For *rad51* mRNA analysis in Fig. S4B (Supporting information), an additional experiment was performed by aging flies 1, 5, 8, 15, and 29 days, and ΔΔC_T_ values for 1‐, 5‐, and 8‐d.o. flies were combined.

### Western blot analysis of protein levels

For each protein extract, three male flies were flash frozen and ground with a pestle in lysis buffer A (50 mm Tris, pH 7.8, 1% NP‐40, 150 mm NaCl) containing protease inhibitors (Complete, Roche), 2.5% 2‐mercaptoethanol, and 1 mM PMSF. A total of 250 U of Benzonase^®^ nuclease was added to each sample, and the mix was incubated for 15–20 min on ice. The soluble lysate was recovered by centrifugation (10 min, 16873 × g 4 °C) and precipitated in acetone before resuspension in loading buffer (Laemmli). Samples were denatured by 5 min at 70 °C before running them on a TGX 4‐12% polyacrylamide gel (Bio‐Rad) and transferred onto nitrocellulose membrane. Anti‐Rad50 (kind gift from M. Gatti, 1:1000), anti‐Smc5 and anti‐Smc6 (SDI, 1:1000 (Chiolo *et al*., 2011)), and anti‐actin (Abcam, ab8224, 1:1000) antibodies were diluted in TBS–Tween 0.1% plus milk 4%. Secondary antibodies were from Thermo Scientific. Quantification of protein level in Fig. 3B was performed by measuring the band intensity of Western blot signals with Fiji, and levels were normalized to actin and then to 1‐d.o. samples.

### Statistical analysis

All statistical analyses were performed using prism 6 (GraphPad Software; La Jolla, CA, USA), with the methods indicated in the legends.

## Funding

This research was supported by the American Federation on Aging Research (J.R.L.), the National Institutes of Health Grant 1R15GM110454‐01 (J.R.L.), the National Institutes of Health Grant R01GM117376 (I.C.), The Rose Hills Foundation (I.C.), and the Edward Mallinckrodt Jr. Foundation (I.C.).

## Author contributions

J.R.L., I.C., L.D., D.M., and H.E. designed experiments; J.R.L., D.M., H.E., F.S., L.D., and C.M.H. performed experiments; J.R.L., D.M., H.E., F.S., L.D., and I.C. analyzed data; E.J.B. provided statistical support; H.S. generated preliminary data that motivated experiments within this study; J.R.L., I.C., L.D., D.M., and H.E. wrote the manuscript.

## Conflict of interest

None declared.

## Supporting information


**Fig. S1** Mu‐2 repair foci persist in older animals.
**Fig. S2** SSA repair is not significantly affected by age.
**Fig. S3** HR repair of constitutively‐induced DSBs increases with age.
**Fig. S4** mRNA levels of early HR components increase in older flies.
**Table S1** HR proportion of total flies from spontaneous events.
