# Erratum

**DOI:** 10.1111/acel.12633

**Published:** 2017-07-12

Delabaere, L., Ertl, H. A., Massey, D. J., Hofley, C. M., Sohail, F., Bienenstock, E. J., Sebastian, H., Chiolo, I. and LaRocque, J. R. (2017), Aging impairs double‐strand break repair by homologous recombination in *Drosophila* germ cells. Aging Cell, 16: 320–328. https://doi.org/10.1111/acel.12556

In the article, “Aging impairs double‐strand break repair by homologous recombination in *Drosophila* germ cells”, the x‐axis in Figure 2d should read as ‘number of γH2Av foci/cell 24h after hs’ rather than ‘number of γH2Av foci/cell 24h after IR’. The correct version of the figure is shown below:

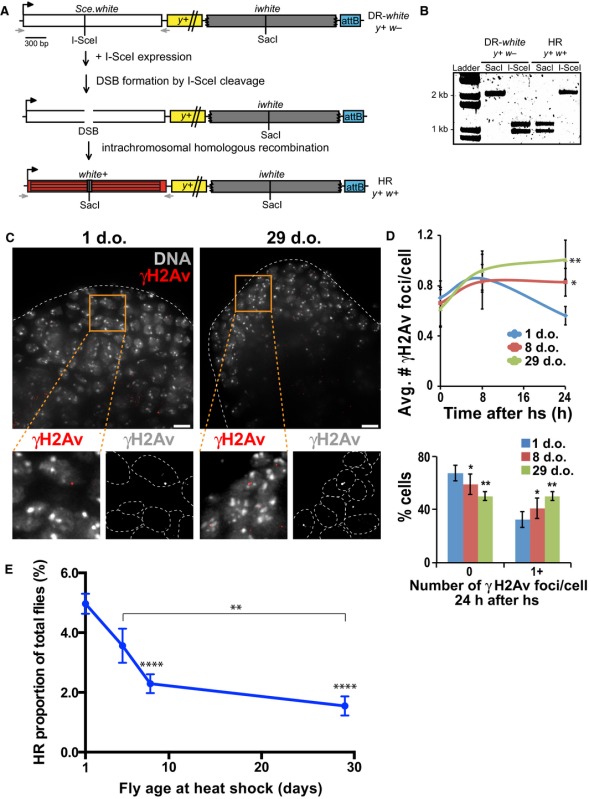

The authors would like to apologize for the inconvenience caused.

