# Supplementary material for: Aging impairs double‐strand break repair by homologous recombination in Drosophila germ cells
Source: Aging Cell. 2016 Dec 21;16(2):320–8. doi: 10.1111/acel.12556 (PMC5334535; doi:10.1111/acel.12556)
Supplement: Supplementary file 1 — Fig. S1 Mu‐2 repair foci persist in older animals. Fig. S2 SSA repair is not significantly affected by age. Fig. S3 HR repair of constitutively‐induced DSBs increases with age. Fig. S4 mRNA levels of early HR components increase in older flies. Table S1 HR proportion of total flies from spontaneous events. [file ACEL-16-320-s001.pdf]

Supporting Information

Aging impairs double-strand break repair by homologous recombination in Drosophila germ cells
Delabaere, *et al.* 2016

Supporting Table 1: HR proportion of total flies from spontaneous events.

Age	Average %HR[a]	SEM	n[b]; total progeny		1d[c]	4d	7d
1d	1.01	0.47	18; 1242		--	--	--
4d	1.03	0.39	11; 679		n.s.	--	--
7d	0.59	0.47	14; 1204		n.s.	n.s.	--
28d	0.46	0.47	11; 681		n.s.	n.s.	n.s.

[a] values based on percentage of red-eyed progeny without heat shock.
[b] n = number of male germlines analyzed; total progeny is all progeny from all germlines scored.
[c] p values based on one-way ANOVA with multiple comparisons, followed by Tukey-Kramer post hoc test.

Supporting Figure Legends

Supporting Figure 1. Mu2 repair foci persist in older animals. Left: IF analysis of premeiotic cells in testes dissected and fixed 8 h after 5 Gy IR shows more γH2Av and mGFP-Mu2/ Mdc1 foci in mitotically dividing spermatogonia of 8 day old (d.o.) flies compared to 1 d.o. flies. Scale bars = 1 μm. Right: Quantification of γH2Av foci and GFP-Mu2/Mdc1 foci in mitotically dividing spermatogonia of 5 and 8-d.o. flies compared to 1 d.o. flies, fixed 8 h after 5 Gy IR. Scale bars = S.D. of at least three independent testes. *$p < 0.05$; **$p < 0.01$; ***$p < 0.001$; ****$p < 0.0001$ (twotailed Mann-Whitney test). Differences in γH2Av foci between 1 and 5 d.o. flies were not significant. n = 87 135 nuclei from at least three independent testes/age/time point.

Supporting Figure 2. SSA repair is not significantly affected by age. (A) DR-*white* flies are crossed with flies containing the hs-I-SceI transgene, which results in DSB formation at the I-SceI recognition sequence. Repair by single-strand annealing (SSA) results in loss of the *yellow* transgene (*y*+) and a yellow-bodied, white-eyed fly (y– w–) in the progeny. (B) Flies containing the DR-*white* reporter and hs-I-SceI transgene were aged to the given times and exposed to heat shock. After 11 days, flies were mated to females and F1 progeny scored for SSA products. Data given is mean ± S.E.M. of 76-123 germlines/age. Differences between the means at each age

were not significant (> 0.05), except for 1 d.o. compared to 5 d.o. (*$p < 0.01$), one-way ANOVA with multiple comparisons, followed by Tukey-Kramer post hoc test.

Supporting Figure 3. HR repair of constitutively-induced DSBs increases with age. Flies containing the DR-*white* reporter and constitutively active I-SceI transgene were crossed to *y w* females at given ages, starting at 1 day old. Every 7 days, flies were mated to new females. F1 progeny of these crosses were scored for HR events. Only data from single male germlines that results in productive vials (≥ 20 progeny) at each time point were analyzed in order to directly compare HR frequencies with age. Average HR frequency and S.E.M are given from 63 germlines. ****$p < 0.0001$ for all comparisons relative to 1 d.o. by one-way ANOVA with multiple comparisons, followed by Tukey-Kramer post hoc test.

Supporting Figure 4. mRNA levels of early HR components increase in older flies. Flies containing the DR-*white* reporter and the hs-I-SceI transgene were aged to the given times and treated as in Figure 3A. (A) C_T values for *rad50, ctip, blm, rad51,* and *rad54* were normalized to that of *rpl32* (C_T) to determine relative expression. $\Delta\Delta C_T$ values were calculated relative to 1 d.o. flies for each gene to determine expression fold change ($2^{-\Delta\Delta CT}$). Averages of the fold change ($2^{-\Delta\Delta CT}$) are given; error bars are S.E.M. of 3-10 biological replicates. ** $p < 0.01$, **** $p < 0.0001$ by one-way ANOVA with multiple comparisons, followed by Tukey-Kramer post hoc test. Comparisons between all other groups were not significant. (B) Male flies containing both DR-*white* and the inducible I-SceI transgene were aged to 1, 5, 8, 15, and 29 days. For 1 and 8 days, the experiment was performed twice and data were combined. For each experiment, two flies were combined to represent one biological replicate and 3-5 biological replicates per age were harvested prior to heat-shock, as described in Experimental procedures. C_T values for *rad51* were normalized to that of *gapdh2* (left) and *rpl32* (right) (C_T) to determine relative expression. $\Delta\Delta C_T$ values were calculated relative to 1 d.o. flies within each experiment for each gene to determine expression fold change ($2^{-\Delta\Delta CT}$). Averages of the fold change ($2^{-\Delta\Delta CT}$) are given and error bars are S.E.M. of 3-10 biological replicates. * $p < 0.05$, ** $p < 0.01$ by ANOVA with multiple comparisons, followed by Tukey-Kramer post hoc test. Comparisons between all other groups were not significant.

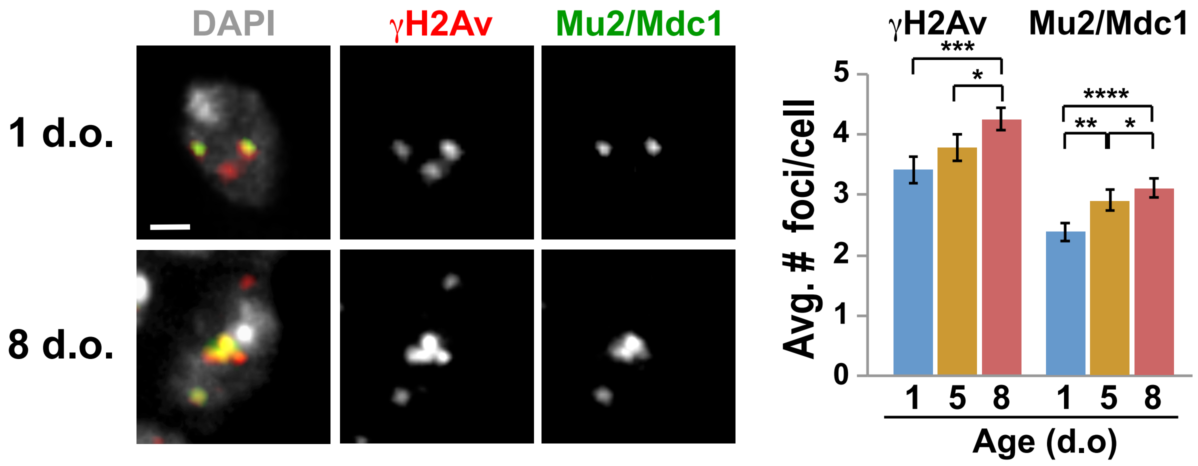

Supplemental Figure 1

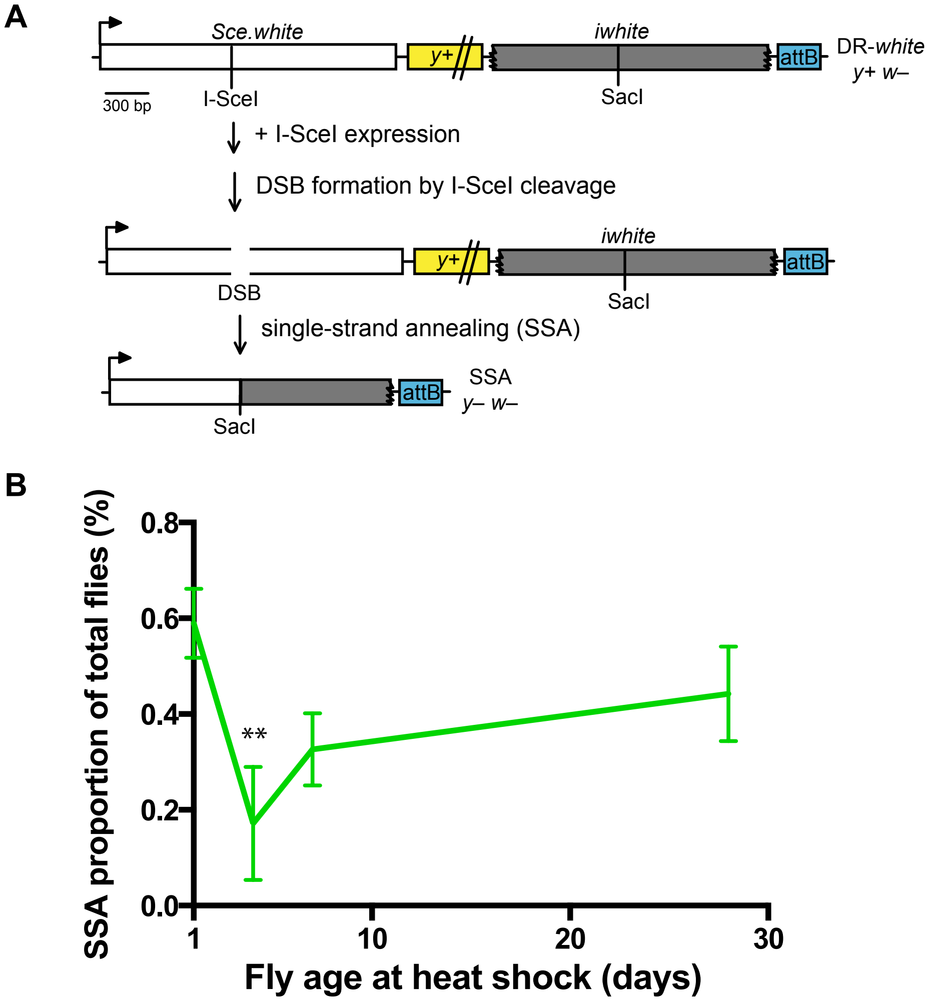

Supporting Figure 2

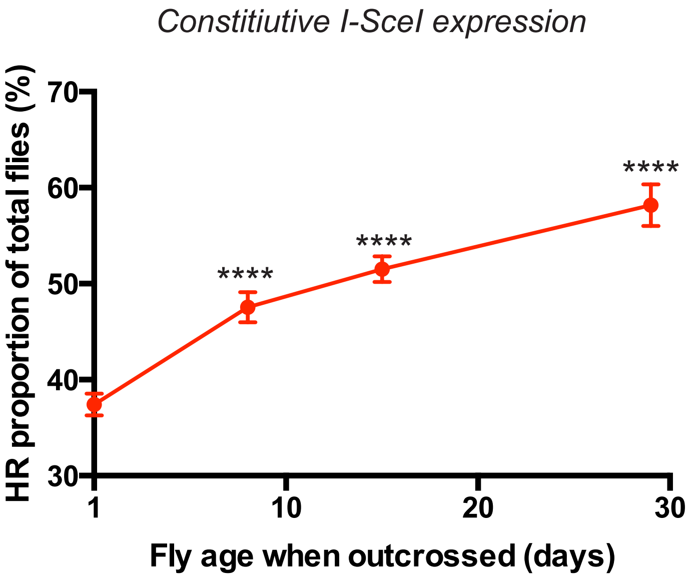

Supporting Figure 3

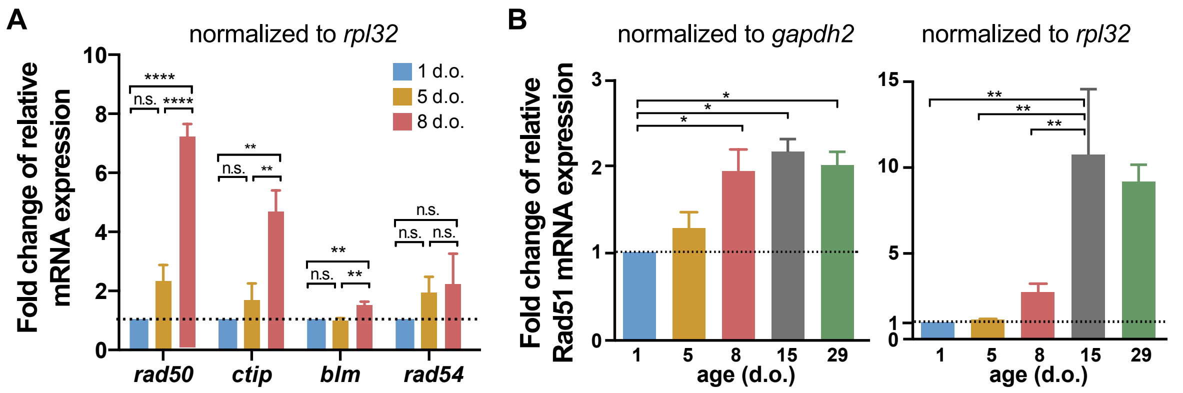

Supporting Figure 4
